# CHLA/ABSC Conference 2023

**DOI:** 10.29173/jchla29678

**Published:** 2023-04-01

**Authors:** Alison Manley, Robin Parker

## Registration is now open for CHLA/ABSC 2023 in Halifax, Nova Scotia!

We are so excited to welcome you back to the East Coast for this year’s conference! Halifax is one of the fastest growing cities in Canada, and a major cultural centre in Atlantic Canada. We are thrilled to be holding the conference on the waterfront in Halifax, which offers great views, easy access, and loads of destinations for eating, shopping, and learning more about the vibrant culture of the Maritimes.

We’ll be kicking off the conference with a welcome reception on Friday, June 2, a chance to get together and catch up before diving into our great program. The following day, we’ll get into our wonderful contributed content, thanks to all of the people who responded to our call for submissions. We also have some excellent keynotes on the docket: Dr. Gaynor Watson-Creed, Associate Dean, Serving and Engaging Society, Faculty of Medicine, Dalhousie University, and Dr. Barbara Hamilton-Hinch, Professor in the Faculty of Health, and Assistant Vice-Provost Equity and Inclusion, Dalhousie University. Both are leading scholars and advocates in the areas in health, medicine, and equity, and we’re looking forward to learning and sharing with them.

In addition to our keynotes and contributed content, we have a great mix of in-person and virtual continuing education sessions, so even if you can’t join us in Halifax, you can still engage in some of the thought-provoking topics we’re focusing on for this year’s conference.

Early bird registration runs until May 6, 2023. You can register for the conference and CE sessions at https://chla-absc.ca/ac_registration.php, and also grab your hotel code from the Accommodations page: https://chla-absc.ca/ac_hotel.php. Follow us on Instagram and Twitter @chlaabscconf to keep up to date with all conference news, and to follow the Haligonian adventures of our whale friend, Putup!

**Alison Manley and Robin Parker**

*2023 Conference Planning Committee Co-Chairs*

## L'inscription est maintenant ouverte pour le congrès ABSC / CHLA 2023 à Halifax, en Nouvelle-Écosse !

Nous sommes réjoui(-e)s de vous accueillir de nouveau sur la côte Est pour le congrès de cette année ! Halifax est l'une des villes à la croissance la plus rapide au Canada et un centre culturel majeur dans le Canada atlantique. Nous sommes ravi(-e)s d'organiser le congrès sur le front de mer d'Halifax, qui offre une vue magnifique, un accès facile et de nombreuses destinations pour manger, faire du magasinage et en apprendre davantage sur la culture vibrante des Maritimes.

La conférence débutera par une réception de bienvenue le vendredi 2 juin, qui sera l'occasion de se réunir et de prendre des nouvelles avant de se plonger dans le programme. Le lendemain, nous aborderons notre merveilleux contenu, grâce à toutes les personnes qui ont répondu à notre appel à contributions. D'excellentes oratrices sont également prévues : Dr Gaynor Watson-Creed, doyenne associée, Servir et engager la société, Faculté de médecine, Université Dalhousie, et Dr Barbara Hamilton-Hinch, professeure à la Faculté de santé et vice-rectrice adjointe Équité et inclusion, Université Dalhousie. Toutes deux sont des chercheures et des défenseures de premier plan dans les domaines de la santé, de la médecine et de l'équité, et nous sommes impatient(-e)s d'apprendre et d'échanger avec eux.

En plus de nos oratrices et de nos contributions, nous proposons un large éventail de sessions de formation continue en personne et virtuelles. Ainsi, même si vous ne pouvez pas vous joindre à nous à Halifax, vous pouvez toujours participer à certains des sujets de réflexion sur lesquels nous nous concentrons pour la conférence de cette année.

L'inscription anticipée est valable jusqu'au 6 mai 2023. Vous pouvez vous inscrire à la conférence et aux sessions de formation continue à l'adresse https://chla-absc.ca/ac_regfr.php?set_lang=french, et obtenir votre code d'hôtel sur la page Hébergement: https://chla-absc.ca/ac_hotel_fr.php?set_lang=french. Suivez-nous sur Instagram et Twitter @chlaabscconf pour vous tenir au courant de toutes les actualités de la conférence, et pour suivre les aventures haligoniennes de notre amie baleine, Putup !

**Alison Manley et Robin Parker**

*Coprésidentes du comité de planification de la conférence 2023*

